# Recognising and managing increased HIV transmission risk in newborns

**DOI:** 10.4102/sajhivmed.v16i1.371

**Published:** 2015-05-20

**Authors:** Max Kroon

**Affiliations:** 1 Division of Neonatal Medicine, Department of Paediatrics, University of Cape Town, South Africa

## Abstract

Prevention of mother-to-child transmission (PMTCT) programmes have improved maternal health outcomes and reduced the incidence of paediatric HIV, resulting in improved child health and survival. Nevertheless, high-risk vertical exposures remain common and are responsible for a high proportion of transmissions. In the absence of antiretrovirals (ARVs), an 8- to 12-hour labour has approximately the same 15% risk of transmission as 18 months of mixed feeding. The intensity of transmission risk is highest during labour and delivery; however, the brevity of this intra-partum period lends itself to post-exposure interventions to reduce such risk. There is good evidence that infant post-exposure prophylaxis (PEP) reduces intra-partum transmission even in the absence of maternal prophylaxis. Recent reports suggest that infant combination ARV prophylaxis (cARP) is more efficient at reducing intra-partum transmission than a single agent in situations of minimal pre-labour prophylaxis. Guidelines from the developed world have incorporated infant cARP for increased-risk scenarios. In contrast, recent guidelines for low-resource settings have rightfully focused on reducing postnatal transmission to preserve the benefits of breastfeeding, but have largely ignored the potential of augmented infant PEP for reducing intra-partum transmissions. Minimal pre-labour prophylaxis, poor adherence in the month prior to delivery, elevated maternal viral load at delivery, spontaneous preterm labour with prolonged rupture of membranes and chorioamnionitis are simple clinical criteria that identify increased intra-partum transmission risk. In these increased-risk scenarios, transmission frequency may be halved by combining nevirapine and zidovudine as a form of boosted infant PEP. This strategy may be important to reduce intra-partum transmissions when PMTCT is suboptimal.

## Introduction

In South Africa, prevention of mother-to-child transmission (PMTCT) programmes have been very successful in reducing the vertical transmission of HIV, with resultant gains in maternal, infant and child health and survival.^1,2^

Complete elimination of mother-to-child transmission (MTCT) remains elusive, primarily because of incomplete programme uptake – owing to suboptimal patient care-seeking behaviour and inadequate health care access – but also because no current antiretroviral therapy (ART) regimen, even when started early in pregnancy, is 100% effective in preventing transmission. Maternal treatment failure resulting from inadequate adherence or drug resistance may also compromise PMTCT programme efficacy. In high-prevalence populations and serodiscordant partners, primary HIV infection in pregnancy (and breastfeeding), after initial negative HIV screening, may contribute disproportionately to transmission.^3^ Delayed ART initiation and apparent incident infection may also be a consequence of false-negative initial testing, owing to procedural and quality issues with rapid antibody screening tests. Finally, resistance to non-nucleoside reverse transcriptase inhibitors (NNRTIs) may compromise the efficacy of infant post-exposure nevirapine (NVP) ‘mono-prophylaxis’.

Although further gains are possible by improving programme coverage and uptake (and this should be encouraged), high-risk vertical exposures at birth remain common and are responsible for a high proportion of transmissions. Recognition of increased-risk scenarios, enhanced labour management (including intra-partum antiretrovirals [ARVs] and caesarean section before labour), infant post-exposure combination ARV prophylaxis (cARP) and a more aggressive testing schedule may all reduce transmission risk and improve the linkage of HIV-infected infants to definitive management. Whilst it is also important for increased risk to be recognised and managed in pregnancy and labour, it is beyond the scope of this article to review obstetric management.

In the developing world, as capacity has grown, PMTCT programmes have evolved from simple, single-dose infant and maternal mono-prophylaxis, focused on intra-partum risk through targeted maternal combination ART (cART), to the universal, lifelong ‘treatment-as-prophylaxis’ defined by the 2013 World Health Organization (WHO) guidelines. Each step in this evolution has resulted in progressive reductions in vertical transmission and improvements in maternal care. Infant prophylaxis has grown from post-exposure prophylaxis (PEP) for intra-partum risk to include post-partum peri-exposure prophylaxis (PEEP) intended to render breastfeeding safer. In the developing world, with the focus on reducing transmission during breastfeeding, little consideration has been given to quantification of the intra-partum risk or to boosting infant PEP for increased intra-partum risk scenarios.

In contrast, developed-world PMTCT guidelines routinely quantify intra-partum risk and recommend boosted infant PEP for increased-risk scenarios. In the event of no or minimal pre-labour prophylaxis, National Institutes of Health guidelines recommend the addition of 3 doses of infant NVP in the first week of life to the standard 6 weeks of infant zidovudine (AZT).^4^ British HIV Association (BHIVA) guidelines recommend triple ARV prophylaxis for infants when the maternal viral load (VL) is > 50 copies/mL, and only recommend AZT mono-therapy if the maternal VL is fully suppressed from 36 weeks’ gestation.^5^ Both guidelines acknowledge a growing body of expert opinion that the benefits of infant post-exposure cARP exceed its risks when transmission risk is increased.

Ironically, pre-2010 South African guidelines provided for routine infant dual prophylaxis with single-dose NVP (sdNVP) and one week of AZT, and infants whose mothers had less than 4 weeks of pre-labour prophylaxis were assigned to sdNVP and 4 weeks of AZT.^6^ This policy incorporated an element of risk recognition and response and was based on findings of the ‘Thai long-short, short-long’ and NVAZ studies.^7,8^

## Quantifying risk

cART initiated early in pregnancy, with a resultant suppressed VL, and avoidance of breastfeeding virtually eliminate vertical transmission. However, even in the developed world, transmissions occur because of suboptimal antenatal care, failure of maternal and infant prophylaxis and primary HIV infection in pregnancy. These factors argue for an augmented approach to infant management in such increased-risk settings. Consequently, guidelines emanating from the developed world have incorporated elements of risk assessment and response.

In the absence of ARVs, HIV transmission rates are approximately 5% – 10% during pregnancy, 10% – 20% during the intra-partum period, and 10% – 20% during extended mixed breastfeeding.^9^ Whilst transmission is minimal in early pregnancy, the frequency of infection increases significantly in the third trimester towards term, and peaks at 10% – 20% during the 8–12 hours of labour and delivery, making the latter the most intense risk period.^10^ The intensity of risk is very low throughout breastfeeding, but the cumulative risk over time may be high. Factors strongly associated with increased transmission risk are: high maternal VL, low maternal CD4 count, the absence of maternal ART, and preterm labour with prolonged rupture of membranes.^11^ Whilst the first three factors are associated with increased transmission in all risk periods, preterm labour, especially with prolonged rupture of membranes, appears only to increase intra-partum transmission risk.

The Women and Infant Transmission Study (WITS) found intra-partum transmission to be associated significantly with a lack of ART, an increased VL, a low CD4 percentage, young gestational age, low birth weight (LBW), prolonged rupture of membranes and hard drug use. Overall transmission, particularly the intra-partum proportion, has declined significantly over time, reflecting the success of peri-partum maternal interventions and infant PEP.^12^ Consequently, the proportion of *in utero* transmissions is higher. In South Africa, the proportion owing to transmission during breastfeeding remains uncertain, as the uptake and duration of breastfeeding are unknown, and long-term follow-up to ascertain postnatal transmission rates has been difficult to achieve.

Intensity of risk varies during pregnancy and breastfeeding according to the timing of ART initiation, the duration of ART and the impact on VL by the time of delivery and breastfeeding. For example, a woman who is newly diagnosed immediately after delivery will be at high risk for transmission in the antenatal and intra-partum periods and will have some risk during early breastfeeding until maternal cART takes effect. In this scenario, infant ARV prophylaxis is critical for reducing transmission during labour and delivery, and exclusive breastfeeding, maternal cART and infant PEEP are vital for risk reduction in the first 3 months of breastfeeding. Heat-treatment of breast milk until an adequate duration of maternal cART or viral suppression has been achieved is an option to eliminate breastfeeding transmission risk entirely. This is especially important to preserve breastfeeding as an option for preterm/LBW infants who are more likely to experience serious adverse events in the first 6 months of life if replacement fed, even if not HIV exposed.^13^

In contrast, a woman who initiates ART from 34 weeks’ gestation, has good adherence, is virally suppressed and delivers at 40 weeks’ gestation will have some risk of transmission during pregnancy, but very little risk during labour and breastfeeding. Whilst a polymerase chain reaction (PCR) test at birth is indicated to detect pre-labour transmissions, infant cARP does not add much benefit and there is no real benefit in extending PEP beyond 6 weeks. It would be wrong to recommend replacement feeding in this case, unless the conditions for safe formula feeding were met.

It is difficult to incorporate this degree of risk assessment nuance into PMTCT programmes, and it may be better to have a binary approach where any non-low-risk scenario, irrespective of risk period, attracts an enhanced response for intra-partum management, infant testing and cARP. The introduction of universal PCR testing at birth would simplify the issue and allow the focus to be solely on flagging increased intra-partum risk exposures for infant cARP. The definition of ’non-low risk’ and extent of the response will need to be balanced against its cost and available resources. If this approach is adopted, then it is important to guard against the perception that all non-low-risk infants should avoid breastfeeding. Many infants in this category are at particularly high risk of formula-feeding-related morbidity and mortality; therefore, exclusive breastfeeding should be encouraged unless the criteria for safe replacement feeding are met.

The community living with HIV has had significant exposure to NNRTIs as part of first-line treatment and as single doses administered during labour. NNRTI resistance develops rapidly after limited exposure, requiring only a single base pair mutation, and vertical transmission of resistant virus has been described.^14,15^ Primary and acquired resistance is not uncommon and, when NNRTI resistance is likely, it seems unwise to rely on NVP mono-therapy for infant PEP when intra-partum risk is increased, even if the mechanism of prophylaxis is different to that of treatment. In contrast, AZT resistance requires numerous mutations and takes longer to develop. Therefore, AZT is important to enhance infant PEP in order to reduce intra-partum transmission risk when maternal virus is likely to be NNRTI resistant.

NNRTI resistance is likely in women who are failing, or have failed, first-line ART, or are on second- or third-line regimens. In such circumstances, it seems logical, at least, to combine infant NVP with AZT if the maternal VL is not suppressed by delivery. The 2013 South African Paediatric Standard Treatment Guidelines recommend expert consultation if resistance is possible, but do not provide advice on reducing the peak risk during labour and delivery with boosted infant PEP.^16^

The following factors are likely to increase vertical transmission risk:

### Maternal factors

- duration of maternal ART < 8 weeks (especially if no pre-labour ART)
- maternal VL > 1000 copies/mL close to term (not always available)
- maternal viral rebound (treatment interruption, poor adherence, true resistance)
- maternal co-morbidity (TB, opportunistic infections, chorioamnionitis)
- incident infection (initial HIV test negative, subsequently tests positive)
- likely NNRTI resistance (second-line ART, failing first-line ART; several sdNVP previously)
- adolescent pregnancy (recent/incident/vertically trans­mitted infection, more likely to have problems with follow-up)
- maternal substance abuse (alcohol or drugs).

### Infant factors

- symptomatic (severe growth restriction, lymphadenopathy, hepatosplenomegaly, thrombocytopaenia, pancytopaenia, congenital cytomegalovirus, congenital syphilis, neonatal tuberculosis)
- preterm delivery (regardless of cause) and/or LBW infants (< 2500 g; < 37 weeks‘ gestation)
- abandoned infants (if Alere Determine or enzyme-linked immunosorbent assay [ELISA] positive).

PCR testing soon after birth will identify infants infected *in utero*, facilitate linkage to definitive care and, hopefully, reduce early mortality. Those not infected *in utero* will, in many instances, also have an increased intra-partum transmission risk and may benefit from enhanced infant PEP. In contrast to the peak risk intensity in labour and delivery, transmission risk intensity per breastfeeding session is extremely low but, owing to a relatively high cumulative risk over time, tends to be overstated. Therefore, the need for enhanced infant PEEP for breast milk exposure is less urgent than PEP for high-risk intra-partum exposure, and the cornerstone of risk reduction in this period remains the urgent optimisation of maternal cART. There is currently no evidence that infant cARP reduces breastfeeding transmission.

## Post-exposure prophylaxis reduces transmission risk

ARV PEP, soon after HIV exposure in various settings, is well established as routine management to prevent transmission:

- In 1997 a case-control study reported that post-exposure AZT mono-prophylaxis reduces transmission in occupational exposures.^17^ Since then, post-exposure cARP has become the standard of care for occupational exposures. Without prophylaxis, the risk of HIV infection from a penetrating injury with an HIV-contaminated needle is estimated at 0.3% compared with an estimated 15% vertical transmission risk in labour.
- Post-exposure cARP is established as the standard of care after sexual assault and after inadvertent exposure of an infant to another mother's HIV-infected breast milk.
- According to Wade et al. in 1998, when AZT was started before 48 hours of life for PMTCT, transmission was 9.3% (4.1% – 17.5%) and, when started on day 3 of life or later, it was 18.4%. Transmission was 26.6% (21.1% – 32.7%) in the absence of AZT.^18^
- A study of HIV-exposed, formula-fed infants whose mothers received no prophylaxis before delivery reported that infant sdNVP or 6 weeks of AZT were equally efficient at reducing vertical transmission. At 6 weeks, transmission was 5.3% with sdNVP and 6.4% with AZT – both considerably less than anticipated without prophylaxis.^19^

Infant prophylaxis probably contributes little to reducing transmission risk when maternal cART is started early and viral suppression is optimal by the last weeks of pregnancy. Extended or augmented infant PEP cannot compensate for suboptimal antenatal prophylaxis, and early maternal ART with VL suppression remains critical to prevent transmission at all times.

Ideally, patients with an increased risk of intra-partum transmission should be flagged for possible elective caesarean section to avoid intra-partum exposure. When maternal prophylaxis is suboptimal, the intra-partum risk peak is a critical opportunity for intervention and, importantly, its relative brevity makes it amenable to infant PEP. In non-breastfeeding settings, infant PEP targets intra-partum risk alone and is the key intervention for reducing intra-partum transmission when there has been no pre-delivery prophylaxis. In breastfeeding settings, infant cARP must include an ARV that reduces breast milk transmission risk (PEEP) as well. Infant AZT treatment has not been shown to be effective in this regard. PEP is usually only effective if initiated within 48–72 hours of delivery. Infant PEP is not effective in reducing *in utero* transmission, but should be commenced as soon as possible after birth to reduce intra-partum transmission.

In most situations, PEP duration is typically 4 weeks but, in the context of PMTCT, AZT for 6 weeks was established as standard by the PACTG 076 study.^20^ In non-breastfeeding settings, there is no real evidence that AZT PEP for 6 weeks is superior to 4 weeks, and the shorter option may be associated with a lower incidence of anaemia and neutropaenia. Pundits for the longer course cite slow decay of transferred intracellular maternal virus as a reason for extending infant PEP.

## Post-exposure combination antiretroviral therapy reduces transmission risk in increased-risk situations

Outside of PMTCT settings, exposures of considerably lower risk (occupational, sexual assault and inadvertent breastmilk exposures) routinely attract cARP. Whilst infant cARP for increased-risk MTCT scenarios is routine in the developed world, little consideration has been given to boosting infant PEP to prevent vertical transmission in the developing world.

If viral suppression by the onset of labour is suboptimal, the risk of intra-partum transmission is increased, and the relatively short duration of labour and delivery makes infant cARP an option for reducing transmissions.

Whilst data to support this approach are limited, there is some direct evidence that infant cARP is more effective than a single agent:

- In infants where maternal pre-labour ARVs were absent or minimal, the addition of 3 doses of infant NVP to the standard 6 weeks of AZT reduced intra-partum transmission from 4.8% (confidence interval [CI] = 3.2–7.1) to 2.2% (CI = 1.2–3.9; *p* = 0.046).^21^
- A study in Malawi by Taha et al. reported that the addition of 1 week of AZT to sdNVP reduced intra-partum transmission from 12.1% to 7.7% (*p* = 0.03).^22^

## Post-exposure combination antiretroviral therapy options and recommendation for South Africa

There are two main potential benefits of cARP:

- reduced intra-partum transmission
- prophylaxis as very early treatment of HIV-infected infants while awaiting PCR results.

A potential disadvantage is consequent difficulty in confirming infection owing to the effect on VL, but this is already an issue with extended NVP mono-therapy in infants. A higher rate of anaemia owing to extended infant AZT argues for restricting the AZT component to 4 weeks.

Selection of appropriate ARVs for combination prophylaxis is problematic as there are limited pharmacokinetic, safety and efficacy data for the use of many agents in the neonatal period, and more so in combination and with preterm babies. Whilst the imperative of early treatment justifies the use of some of these agents in neonatal cART for newborns infected *in utero*, it is more difficult to justify their use as cARP in HIV-exposed neonates who, for the most part, remain uninfected.

The 2008 South African PMTCT programme recommended sdNVP and AZT extended to 1 or 4 weeks. Since 2010, extended infant daily NVP up to 12 months has been used as PEP and breastfeeding prophylaxis.

AZT, lamivudine (3TC) and NVP as infant prophylaxis are the most used and studied agents globally. There are good study data on the safety and efficacy of these agents as part of comprehensive PMTCT regimens.^23,24,25^ BHIVA guidelines recommend these three drugs in combination for infant PEP when transmission risk is not minimal. If drug resistance is likely, consideration needs to be given to other agents not usually used for infant PEP, and then only under expert guidance and preferably with resistance genotyping.^26^

Lopinavir/ritonavir (LPV/r) has been associated with very significant toxicity and is not recommended for use before 42 weeks’ corrected gestational age, limiting its usefulness as an option in cARP.^27^ Where multidrug resistance is identified by maternal virus genotyping prior to delivery, serious consideration may need to be given to LPV/r use as infant cARP, but then only in a strictly controlled environment.

As extended infant NVP facilitates safer breastfeeding by HIV-infected infants, it makes sense to retain NVP as the core of infant prophylaxis and add 4 or 6 weeks of AZT and possibly 3TC. There is no evidence that extending AZT or 3TC beyond 6 weeks confers any further intra-partum transmission reduction, or that it affects breastfeeding risk. Both LPV/r and 3TC as single agents extended to 12 months of breastfeeding have recently been reported in the PROMISE PEP study to reduce HIV transmission during breastfeeding to very low levels.^28^

Currently, in South Africa, the two main options for cARP are for a two- (AZT, NVP) or three-drug (AZT, 3TC, NVP) approach. There is little evidence to support one over the other and, indeed, the three-drug approach in HPTN040 showed no clear benefit over the two-drug approach.

Dosage tables for NVP (Table 1) and AZT (Table 2) are given below. These tables are modified from the Western Cape 2014 PMTCT Guidelines, include doses for preterm infants, and differ slightly from doses provided in the current National Guidelines.

**TABLE 1:** Nevirapine doses for post-exposure prophylaxis in the first 6 weeks of life.

Birth weight	Age	Daily dosage	Daily volume
< 2.0 kg	Birth – 2 weeks	2 mg/kg	0.2 mL/kg
	2–6 weeks	4 mg/kg	0.4 mL/kg
2.0 kg – 2.5 kg	Birth – 6 weeks	10 mg	1 mL
> 2.5 kg	Birth – 6 weeks	15 mg	1.5 mL

Note: Nevirapine syrup (10 mg/mL).

**TABLE 2:** Zidovudine doses for post-exposure prophylaxis.

Birth weight/gestational age	Age	Dosage	Dose volume
< 2 kg and > 35 weeks	Birth – 4 weeks	4 mg/kg/dose 12-hourly	0.4 mL/kg/dose 12-hourly
> 2 kg and > 35 weeks	Birth – 4 weeks	12 mg 12-hourly	1.2 mL 12-hourly
If gestational age 30–35 weeks	Birth – 2 weeks	2 mg/kg/dose 12-hourly	0.2 mL/kg/dose 12-hourly
	2–4 weeks	3 mg/kg/dose 12-hourly	0.3 mL/kg/dose 12-hourly
If gestational age < 30 weeks	Birth – 4 weeks	2 mg/kg/dose 12-hourly	0.2 mL/kg/dose 12-hourly

Note: Zidovudine syrup (10 mg/mL).

The recommended dosage for 3TC is 2 mg/kg for the first 4 weeks of life, irrespective of gestational age at birth.

## Conclusion

The most intense period of risk for MTCT is during labour and delivery. This is accentuated by suboptimal maternal viral suppression and limited pre-labour cART duration.

An increased risk of intra-partum HIV infection can be reduced by boosted infant PEP. Newborns at increased risk can be identified by clinical and laboratory parameters at birth, be tested early, and receive post-exposure cARP if the birth PCR test is negative.

Risk of transmission during breastfeeding is low but, because of the cumulative risk over the whole breastfeeding period, tends to be overstated. Breastfeeding risk per month is very low with maternal cART and infant PEEP. In high-risk scenarios, maternal ART/adherence must be optimised urgently to reduce breastfeeding transmission risk and improve maternal health and survival. There are no data on the efficacy of cARP to prevent breastfeeding transmission of HIV in high-risk situations, and the potential toxicity of extended cARP means it cannot be recommended for this purpose.

PCR testing at birth is recommended for infants commencing cARP and when *in utero* transmission risk is high, to respectively exclude HIV infection and facilitate prompt linkage of infected infants to definitive treatment.

Appendix 1 contains commentary on the new South African *Consolidated guidelines for PMTCT and the management of HIV in children, adolescents and adults* that were released after the writing of this article.

## Appendix 1: Recognising and managing increased HIV transmission risk in newborns

In January 2015, the South African National Department of Health (NDoH) issued the new *Consolidated guidelines for PMTCT and the management of HIV in children, adolescents and adults*. This document includes risk criteria linked to more intensive testing and prophylaxis regimens.

Immediate PCR testing including birth testing is now recommended for many increased risk situations.

Infant post-exposure cARP with AZT and NVP for increased intra-partum risk is recommended for 6 weeks when the most recent maternal VLs are > 1000 copies/mL. If maternal ART duration is < 4 weeks or the diagnosis of maternal HIV is made during labour or after delivery, infant breastfeeding PEEP with NVP as a single agent is extended to 12 weeks.

Unfortunately, cARP is not recommended for the very situation where evidence for risk reduction is strongest: when there is no pre-labour ART and a current VL result is not available. Another important omission is the use of cARP for suspected maternal virus NNRTI resistance in instances where it does not make sense to rely solely on NVP for prevention.

Curiously, maternal seroconversion during breastfeeding does attract temporary cARP with AZT for 1 week and extended NVP, but this advice seems illogical and the evidence for benefit is lacking.

Unlike the 2013 WHO consolidated guidelines, the NDoH guidelines make no mention of ARV prophylaxis dosing for preterm and LBW infants. The NVP dosing schedule recommends 20 mg for all infants > 6 weeks old. Many preterm infants may weigh < 2 kg at 6 weeks, and the 20 mg dose may be too high. Moreover, the simplified AZT dosing schedule does not include recommendations for infants weighing < 2 kg.

Whilst the consolidated guidelines are a step in the right direction, they could be improved further, and clinicians should carefully consider whether patients with increased-risk intra-partum HIV exposure may benefit from enhanced prophylaxis. The adoption of universal birth PCR testing would simplify risk management and the selection of infants for cARP. Point-of-care nucleic acid testing in delivery facilities has great potential for routine maternal VL testing to determine intra-partum transmission risk and to facilitate universal birth PCR testing for HIV-exposed newborns.
